# Weight Regain After Sleeve Gastrectomy: A Look at the Benefits of Re-sleeve

**DOI:** 10.7759/cureus.3450

**Published:** 2018-10-14

**Authors:** Christian Saliba, Johnny El Rayes, Samer Diab, Gregory Nicolas, Raja Wakim

**Affiliations:** 1 General Surgery, Lebanese American University-Medical Center, Beirut, LBN; 2 Orthopedics, Saint Joseph University, Beirut, LBN; 3 General Surgery, Mount Lebanon Hospital, Beirut, LBN

**Keywords:** revision laparoscopic sleeve gastrectomy, residual fundus, weight regain, estimated weight loss

## Abstract

Introduction

Laparoscopic sleeve gastrectomy (LSG) has become one of the most commonly performed weight loss procedures due to its simpler technique and lower complication rate as compared to the Roux-en-Y gastric bypass and duodenal switch. However, weight regain is seen in patients with a large gastric fundus. In these cases, a revision laparoscopic sleeve gastrectomy (reLSG) aiming at resecting the excess pouch is a promising option for correction.

Methods

From April 2013 to March 2016, six patients underwent a reLSG for a failure of weight loss after the demonstration of a large gastric fundus on the upper gastrointestinal (UGI) series.

Results

One patient out of six (16.7%) suffered from a gastric leak and was lost to subsequent follow-up. The rest (83.3%) had a smooth recovery and were followed up for a mean of 18 months. Mean excess weight loss (EWL) was 68%, with a minimum of 48% and a maximum of 75%.

Conclusion

reLSG is a promising option for failed weight loss after LSG in patients who demonstrate the presence of a large gastric pouch. It carries a higher complication rate than the initial procedure. Further trials and meta-analyses are needed to prove the efficacy of this procedure.

## Introduction

Laparoscopic sleeve gastrectomy (LSG) emerged as a new approach to bariatric surgery and has been increasing in popularity to become one of the most commonly performed weight loss procedures. Its relatively simple technique and low complication rate contributed to it being preferred over older surgical techniques: Roux en Y gastric bypass (RYGB) and laparoscopic duodenal switch (DS) [1-2].

However, in up to 30% of the cases, a revision surgery is required for reasons that include inadequate weight loss, weight regain, and/or the development of severe upper gastrointestinal symptoms [3]. Weiner et al. described two mechanisms of weight regain post LSG: loss of restriction and/or changes in eating behavior [4]. Further explanations have been speculated, which include technical errors, such as false calibration with a large bougie and incomplete section of the gastric fundus, as well as physiologic changes, such as dilation of the residual stomach [4].

Traditionally, a conversion to DS or more commonly, to an RYGB has been the standard of care [5]. However, the discovery of a possible dilation of the residual stomach or the presence of a remaining gastric fundus led to changes in the approach of a failed LSG and the practice of a revision LSG (reLSG) emerged with the rationale of resizing the sleeve when dilation is present on an upper gastrointestinal (UGI) series or a computerized tomography (CT) scan volumetry [5-6].

The aim of this study is to evaluate the efficacy and the complications of a reLSG in cases of a residual fundus.

## Materials and methods

**Figure 1:**
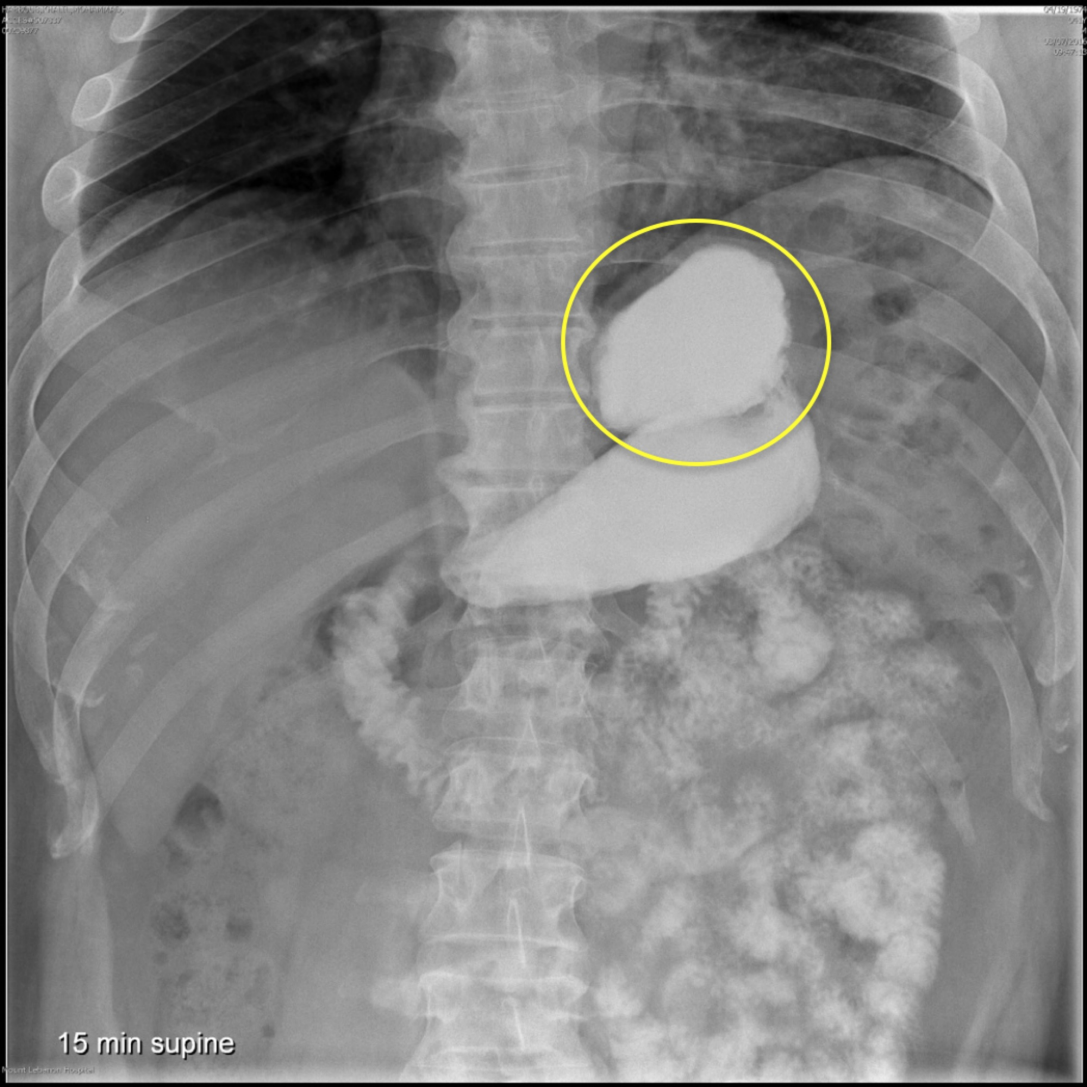
Upper GI series revealing the presence of a dilated fundus (yellow circle) GI: gastrointestinal

Between April 2013 and March 2016, we reviewed six patients who failed an initial LSG (defined as excess weight loss of less than 50% or weight regain after 18 months). A UGI series was performed and revealed the presence of a dilated fundus in all subjects (Figure 1). They were thus candidates for a reLSG. It should be noted that computed tomography (CT) scan volumetry is not available at our center. Mean follow-up was 18 months following the operation and the results were compared with those of their initial operation so as to evaluate the efficacy of the reLSG.

Surgical technique

For our reLSG, we opted for the same operative technique as the initial operation. Starting with a laparoscopic investigation of the abdominal cavity, we discovered multiple adhesions in the gastro-hepatic window and between the stomach and the gastro-splenic ligament. Adhesiolysis was performed for the liberation of the greater curvature. The hiatus was subsequently dissected and the neofundus was identified. A laparoscopic mechanical stapler, size 60, was used to cut the fundus. In this case, due to an increased risk of leak, we used black cartridges. An intraoperative methylene blue test was performed and showed no immediate signs of gastric leak. A gravity drain was left in the epigastric cavity. Mean operation time was 93 minutes. A clear liquid diet was started on postoperative day one and patients were discharged on day two.

Postoperative management

Patients had no complications postoperatively, except for one out of six patients (16.7%) who suffered from a leak that appeared 15 days after the surgery. This patient had a drain inserted in the epigastric area, next to the fundal leak, by an interventional radiologist and was subsequently kept without enteral nutrition. The rest (83.3%) had a smooth recovery and were discharged on postoperative day two.

## Results

Our results are based on a mean follow-up of 18 months for five patients (Table 1). There were four women and two men. Mean age was 48.7 years. One female patient suffered from a fundal leak and was lost to subsequent follow-up. Mean excess weight loss (EWL) was 64%, with a minimum of 48% and a maximum of 75%. 

**Table 1:** BMI values before LSG, interval time, BMI values before and after re-sleeve for six patients BMI: body mass index; LSG: laparoscopic sleeve gastrectomy

	BMI before LSG (Kg/m^2^)	Interval time (months)	BMI before re-sleeve	Interval time (months)	BMI after re-sleeve (Kg/m^2^)	Complication
Patient 1	43.4	36	41.7	12	34	-
Patient 2	52.4	12	43.2	10	33	-
Patient 3	37.6	55	33	15	28	-
Patient 4	47.6	30	40	10	37	-
Patient 5	53	28	38.5	48	32	
Patient 6	44	34	41	-	-	Leak

## Discussion

Laparoscopic sleeve gastrectomy (LSG) has been validated as being an appropriate bariatric operation for weight loss in obese patients and in the management of obesity for associated cardiovascular comorbidities [7]. Himpens et al. stated in a long-term report of laparoscopic sleeve gastrectomy that after six years, the EWL exceeds 50% [8]. It offers the advantages of weight loss and less technical demand without the disadvantages of malabsorptive techniques (DS and RYGB). These procedures have a higher learning curve due to multiple bypassing anastomoses [9]. The malabsorptive procedures are also more morbid for patients due to disadvantages in terms of vitamins, oligo minerals deficiencies, and distorted digestive tract causing any future endoscopic exploration to be futile [9].

As with all bariatric surgeries, LSG is also associated with long-term weight regain. A recent systematic review has shown that the rate of weight regain could range from 5.7 % at two years to 75.6 % at six years [10]. Multiple explanations were speculated regarding the failure of LSG but the one that has taken a big interest in the literature is the large gastric size found in these patients. The theory of a physiologic dilation of the remnant stomach over time is a source of debate in the literature [2]. Braghetto et al. reported a mean gastric volume increase from 108 to 250 ml, measured by CT scan volumetry, from postoperative day three to 24-36 months after surgery [11]. Another theory is the initial malresection of the gastric fundus. Noel et al. described that in many cases, the dissection over the fundus, especially on the posterior aspect, may be difficult and missed, and stated that the success of LSG is learning curve dependent [5]. Similarly, Lannelli et al. reported that the incomplete removal of the gastric fundus seems to be the most reliable hypothesis for a remnant pouch [2]. It may be technically demanding and almost impossible in some patients, notably the extremely obese.

Based on radiological studies by Braghetto et al., a threshold of 250 cm^3^ measured by CT-scan volumetry has been proposed as a possible indication for reLSG, also termed fundectomy, while a residual volume below this threshold encourages the conversion to a malabsorptive procedure [11]. This speculation has been adapted and further encouraged by others [5-6,12].

Other surgical options are available for weight regain following LSG, the most common being DS and RYGB. DS is appropriate if the original operation was set to be the first part of the whole operation. The Roux en Y gastric bypass is appropriate if following LSG, the patient develops symptoms of severe gastroesophageal reflux disease. However, these two operations are accompanied by a higher rate of postoperative complications as compared to a reLSG. In a series comparing 59 DS with 88 LSG, Topart reported that the complication rate was higher after DS than after reLSG [13]. This re-intervention offers several advantages, compared with the malabsorptive procedures, such as increasing restriction and decreasing gastric output, lesser dumping syndrome by preserving the pylorus, decreased risks of anemia, osteoporosis, protein and vitamin deficiency (except B12 and thiamine levels), and shorter operative time [2].

In our study, we reviewed six patients who underwent reLSG due to either insufficient weight loss after 18 months post LSG or progressive weight regain. Many factors exist that could lead to these outcomes (noncompliance with a proper diet, increased ghrelin levels, inadequate follow-up support, maladaptive lifestyle behaviors, etc.) [10]. We focused on the increased fundus size post LSG either de novo or by progressive dilatation. Consequently, every patient with insufficient weight loss on subsequent clinical follow-up was subjected to a UGI study. The latter showed an overly large fundus as the most likely implicated factor in the failure of weight loss.

As mentioned before, from the six patients we had, one patient suffered from a gastric leak next to the fundal staple line. This shows that our risk of leak complications after a reLSG was at a 16.7%, higher than the risk rate of the initial operation. This percentage was also similar to other series, ranging from 10.2% to 14.5% [3,6]. The rest of the patients registered a mean EWL of 64% with ranges from 48% to 75% after a mean follow-up of 18 months. One patient persevered with a proper diet regimen with exercise and maintained an EWL of 75% after 48 months of follow-up.

Our numbers (EWL=64%) were very encouraging and similar to the numbers achieved from certain previous studies and even higher than some. Noel et al. achieved an EWL of 58.7% at a mean follow-up of 19.9 months [5]. Rebibo et al. achieved 66.5% after 12 months of follow-up [6]. Silecchia et al. achieved 53.4% at 24 months of follow-up [3]. Lannelli et al. showed an EWL of 46.5% after a follow-up of 27.7 months [2]. Our results were satisfying. However, the small population diminishes the power of our study. The patient postoperative follow-up was not uniform, with some patients refusing to change their previous lifestyle. This was noted by an EWL ranging from 48% to 75%. The patient with an EWL of 48% was not willing to follow a proper diet and not willing to adhere to an exercise regimen.

## Conclusions

Laparoscopic sleeve gastrectomy is an approved bariatric surgery able to achieve a significant EWL of 55% up to two years of follow-up. However, many regain their lost weight in up to five years following the index surgery. This is mainly due to patient noncompliance with a proper diet and exercise regiments and to surgery-related factors in the formation/keeping of a neofundus. A revision LSG is a promising new technique in selected patients with a large fundus on subsequent UGI series. It offers significant weight loss and an EWL of 64% following our study with a complication rate (fundal leak) of 16.7%. The results of our study were almost similar to the results of previous similar studies; patients with a neofundus will benefit from a reLSG in terms of EWL. However, our results were weakened in impact by the low population. Randomized controlled trials and systematic reviews about this subject are needed in order to induct this management plan in the current surgical guidelines of weight regain management following LSG.

## References

[1] Spiegel HU, Skawran S (2011). From longitudinal gastric resection to sleeve gastrectomy—revival of a previously established surgical procedure. J Gastrointest Surg.

[2] Lannelli A, Schneck AS, Noel P, Ben Amor I, Krawczykowski D, Gugenheim J (2011). Re-sleeve gastrectomy for failed laparoscopic sleeve gastrectomy: a feasibility study. Obes Surg.

[3] Silecchia G, De Angelis F, Rizzello M, Albanese A, Longo F, Foletto M (2015). Residual fundus or neofundus after laparoscopic sleeve gastrectomy: is fundectomy safe and effective as revision surgery?. Surg Endosc.

[4] Weiner RA, Weiner S, Pomhoff I, Jacobi C, Makarewicz W, Weigand G (2007). Laparoscopic sleeve gastrectomy--influence of sleeve size and resected gastric volume. Obes Surg.

[5] Noel P, Nedelcu M, Nocca D, Schneck A, Gugenheim J, Iannelli A, Gagner M (2013). Revised sleeve gastrectomy: another option for weight loss failure after sleeve gastrectomy. Surg Endosc.

[6] Rebibo L, Fuks D, Verhaeghe P, Deguines JB, Dhahri A, Regimbeau JM (2012). Repeat sleeve gastrectomy compared with primary sleeve gastrectomy: a single-center, matched case study. Obes Surg.

[7] Fezzi M, Kolotkin RL, Nedelcu M (2011). Improvement in quality of life after laparoscopic sleeve gastrectomy. Obes Surg.

[8] Himpens J, Dobbeleir J, Peeters G (2010). Long-term results of laparoscopic sleeve gastrectomy for obesity. Ann Surg.

[9] Regan JP, Inabnet WB, Gagner M, Pomp A (2003). Early experience with two-stage laparoscopic Roux-en-Y Gastric bypass as an alternative in the super-super obese patient. Obes Surg.

[10] Lauti M, Kularatna M, Hill AG, MacCormick AD (2016). Weight regain following sleeve gastrectomy—a systematic review. Obes Surg.

[11] Braghetto I, Davanzo C, Korn O (2009). Scintigraphic evaluation of gastric emptying in obese patients submitted to sleeve gastrectomy compared to normal subjects. Obes Surg.

[12] Deguines JB, Verhaeghe P, Yzet T, Robert B, Cosse C, Regimbeau JM (2013). Is the residual gastric volume after laparoscopic sleeve gastrectomy an objective criterion for adapting the treatment strategy after failure?. Surg Obes Relat Dis.

[13] Topart P (2011). Comment on: laparoscopic repeat sleeve gastrectomy versus duodenal switch after isolated sleeve gastrectomy for obesity. Surg Obes Relat Dis.

